# Does the Impact of the Tobacco Epidemic Explain Structural Changes in the Decline of Mortality?

**DOI:** 10.1007/s10680-016-9384-2

**Published:** 2016-08-22

**Authors:** F. Peters, J. P. Mackenbach, W. J. Nusselder

**Affiliations:** 1 Department of Public Health, University Medical Center, Rotterdam, The Netherlands; 2 Institute of Sociology and Demography, University of Rostock, Ulmenstrasse 69, 18057 Rostock, Germany

**Keywords:** Tobacco epidemic, Smoking, Life expectancy, Projection, Structural change, Breakpoint

## Abstract

Since 1950, most developed countries have exhibited structural changes in mortality decline. This complicates extrapolative forecasts, such as the commonly used Lee–Carter model, that require the presence of a steady long-term trend. This study tests whether the impact of the tobacco epidemic explains the structural changes in mortality decline, as it is presumed in earlier studies. For this purpose, the time index of the Lee-Carter model in males was investigated in 20 developed countries between 1950 and 2011 for possible structural changes. It was found that removing the impact of smoking from mortality trends took away more than half of the 12 detected trend breaks. For the remaining trend breaks, adjusting for smoking attenuated the degree of change in mortality decline. Taking the tobacco epidemic into account should become standard procedure in mortality forecasts to avoid a misleading extrapolation of trends. Nevertheless, more research is needed to identify additional factors, such as health-care policies and innovations in medical treatment, to explain the remaining structural changes.

## Introduction

Nowadays, there is increasing interest in more accurate projections of future mortality and life expectancy (Pitacco et al. 2009; De Waegenaere et al. 2010). In the past, official projections and expert appraisals were often too pessimistic, failed to foresee the huge improvements in life expectancy, and often underestimated the potential for a further decline in mortality (Stoeldraijer et al. 2013; Cruijsen and Eding 2001; Ahlburg and Vaupel 1990; Keilman 2008). This failure to provide reliable projections of mortality rates can jeopardize future pension plans, life annuities and the public provision of health insurance (Mayhew and Smith 2011; Christensen et al. 2009; Pitacco et al. 2009).

In response to the recurrent failure to accurately project mortality trends, new models have been proposed (Booth and Tickle 2008; Wong-Fupuy and Haberman 2004; Cairns et al. 2009; Girosi and King 2008). These new approaches have in common that they extrapolate mortality trends using only past series of mortality rates as input in their model (Booth and Tickle 2008; Lee and Carter 1992). This displaces earlier approaches that also incorporated external information such as biological reasoning, expert knowledge and information on causes of death, which often proved to make projections worse rather than better (Wilmoth 1995; Tabeau et al. 2001).

One prominent extrapolation approach is the Lee–Carter model (LC model) and its variants, often referred to as the ‘gold standard’ or ‘benchmark’ (Lee and Carter 1992; Buettner 2002; Li and Chan 2007). Besides the model’s parsimony and the intuitive interpretation of its parameters, the major reason for its success is its congruence with historic trends. In their seminal paper, Lee and Carter (1992) demonstrated that the central mortality time trend in the USA between 1900 and 1989 could best be described by a simple straight line, which provides a solid basis for further linear extrapolation of the decline in mortality. This remarkable regularity of the decline in mortality rates has been confirmed for many other high-income countries, so that linear extrapolation became the leading paradigm of mortality projections (Tuljapurkar et al. 2000; Oeppen and Vaupel 2002; White 2002; Booth and Tickle 2008).

However, past mortality trends were not linear in all countries and subpopulations. An increasing number of countries exhibited more irregular mortality trends in the past decades, including the Netherlands, Norway, Denmark, Australia and the USA (Nusselder and Mackenbach 2000; Juel et al. 2000; Meslé and Vallin 2006; Mackenbach et al. 2011; Booth et al. 2002). A recent series of papers even reported the presence of significant structural breaks in the time index of the LC model in almost any high-income country (O’Hare and Li 2014b; Coelho and Nunes 2011; Li et al. 2011). Thereby, most of the breaks occurred in males and during the 1970s and 1980s (Coelho and Nunes 2011; Li et al. 2011; O’Hare and Li 2014b; Ouellette et al. 2014). In the presence of such nonlinearity, past trends do not provide a solid basis for extrapolation and the resulting projections become more volatile and particularly sensitive to the selection of the historical period (Peters et al. 2012; Janssen and Kunst 2007). To develop better projection models, knowledge on the underlying determinants responsible for the structural breaks is required.

One possible explanation for the presence of structural breaks in mortality trends is the distorting impact of the progression of the tobacco epidemic that affected male mortality trends mainly during the 1970s and 1980s, i.e., the periods in which many of the structural breaks were detected (Janssen et al. 2013; Thun et al. 2012). Thereby, the impact of the tobacco epidemic on mortality trends showed a regular bell-shaped pattern following the trend in smoking prevalence with a time lag of about 20–30 years (Thun et al. 2012; Lopez et al. 1994). Hence, the slowing down and acceleration in the pace of mortality decline might be a result of the different stages of progression of the impact of the tobacco epidemic on mortality (Lopez et al. 1994). After adjusting for the distorting effects of smoking, trends in male life expectancy were more linear over time, more similar between the countries, and closer to the already more linear trends of females (Bongaarts 2006; Janssen and Kunst 2007). Further, a recent study documented that the smoking prevalence in 10 countries was negatively correlated with both the compression of the age-at-death distribution and the delay of aging (Janssen et al. 2015).

Until now, most projection models ignored the factor ‘smoking’, despite its dramatic impact on mortality trends. The overview and quantitative comparison of models in the reports of Shang et al. (2011); Dowd et al. (2010); Cairns et al. (2009) do not cover the topic of smoking at all. However, a recent explorative study by Kleinow and Cairns (2013) on the link between smoking prevalence and mortality is a first indication of the awareness of this factor in the actuarial field. More recently, demographers have developed projection models that take smoking into account by including data on the intensity of smoking either directly from health surveys or indirectly from observed trends in lung cancer (King and Soneji 2011; Janssen et al. 2013; Wang and Preston 2009). Nevertheless, it is unknown in which cases these models should be preferred to simpler models not taking into account the impact of smoking, and whether other factors behind the irregular mortality trend might have been overlooked.

To fill this gap in knowledge, the present study examines to what extent structural breaks in the central time trend of the LC model in high-income countries were indeed caused by the impact of smoking. Thereby, we focus on male mortality trends, where the impact of the smoking epidemic already peaked in most countries, while it is generally still increasing among females, so that structural changes due to smoking might occur more likely in the future. If smoking indeed explained most of the breaks in males, projection models including information on smoking should become a standard approach in mortality forecasting. If this was not the case, then researchers should aim to identify other factors that additionally explain irregular mortality trends.

## Data and Methods

### Data

The impact of smoking on trends in male mortality was tested using data for the period 1950–2011 from 20 OECD countries: Australia, Austria, Belgium, Canada, Denmark, Spain, Finland, France, Scotland, England & Wales, Northern Ireland, Ireland, Italy, Japan, the Netherlands, Norway, Portugal, Sweden, Switzerland and the USA. These countries were selected because reliable information on lung-cancer mortality and all-cause mortality could be obtained for almost the full time span from harmonized sources. Eastern European countries were not included because structural changes probably occurred in response to the fall of the Iron Curtain around 1990, and also because data for these countries were not fully available and/or reliable for the full period. Different parts of the UK were analyzed separately because large spatial variation has been reported with respect to mortality trends in the UK different areas (Murray 1967). We did not include smaller countries (such as Iceland and Luxembourg), where irregularities in time trends might be due to the small number of events rather than to external influences.

To derive mortality rates and construct life tables, we used age-specific population counts and death counts from the Human Mortality Database in the age groups (0, 1–4, 5–9, …, 80–84, 85+ years) (Human Mortality Database 2015). Further, we obtained sex and age-specific lung-cancer death rates for the age groups (35–40, …, 80–84, 85+ years) from the WHO mortality database on causes of death, as input for the indirect estimation of smoking-attributable mortality (World Health Organization 2015). We do use the full age range between age 0 and 85+ in our main analysis as this age range is the basis for computing life expectancy at birth that is of main interest for many applications, but checked the sensitivity of our results if the sample was restricted to more narrow age ranges.

## Methods: Fitting the LC model

To extract a central time trend of mortality, we applied the LC model as expressed in Eq. (1) (Lee and Carter 1992). In this model, the average log mortality rate at each age *α*_*x*_ is separated from the central time trend *κ*_*t*_ while allowing for slower and faster rates of decline at every age through the interaction term *β*_*x*_

1 $$\log m_{x,t} = \alpha_{x} + \beta_{x} \kappa_{t} + \varepsilon_{x,t}$$

To fit the LC model introduced in (1), we followed Brouhns et al. (2002) who assumed that deaths were drawn from a Poisson distribution with person-years lived as offset and estimated the LC parameters via maximum likelihood (Brouhns et al. 2002). This provided a more realistic assumption for the variance in death rates (Pitacco et al. 2009). To achieve a unique solution of model (1), the following restrictions were made, which is in line with earlier studies (Cairns et al. 2009):

2 $$\mathop \sum \limits_{t} \kappa_{t} = 0$$

and

3 $$\sum_{x} \beta_{x} = 1$$

This means that the sum of all values of the time index *κ*_*t*_ adds up to zero so that the age profile of mortality rates, represented by α_*x*_, represents the average profile of all years *t*. The sum of *β*_*x*_ was scaled to one to ensure that *κ*_*t*_ and *β*_*x*_ could be identified uniquely, which neither affects the fit of the model nor the projection of mortality rates. The model was fitted in *R* using the *lifemetrics* package (available at: http://www.macs.hw.ac.uk/~andrewc/lifemetrics/). We fitted the LC model to all-cause mortality rates and to mortality rates where the impact of smoking was removed beforehand, as described below.

## Methods: Estimating the Impact of Smoking on Mortality

We applied the indirect approach suggested by Preston et al. (the PGW approach) (2010) to estimate the fraction of mortality attributable to smoking. Here, the basic idea is that the total cumulative damage of past smoking on all causes of death could be indirectly inferred from observed lung-cancer mortality rates. Defining smoking as the only source of variation in lung-cancer rates [*M*_*L*_], the intensity of smoking is computed as the difference between the observed *M*_*L*_ and the *M*_*L*_ among never smokers at the same age and sex obtained from the Cancer Prevention Study II—a large cohort study with more than one million participants with 6 years of mortality follow-up between 1982 and 1988 (Thun et al. 1982). To obtain the fraction attributable to smoking for causes other than lung cancer [*M*_*0*_], the computed intensity of smoking is multiplied by a sex, age and time-specific translation factor (Preston et al. 2010). These factors were obtained by regressing *M*_*L*_ on *M*_*0*_ for a group of high-income countries between 1950 and 2006. Since the original approach provided translation factors only above the age of 50 years, we also used the additional factors computed by Martikainen et al. (2014) to estimate smoking-attributable mortality between the age of 35–50 years. Additionally, for a few calendar years where no information on lung cancer was available, we linearly interpolated or extrapolated the attributable fractions transformed by the natural logarithm beforehand.

## Methods: Detecting and Dating Structural Changes

To detect structural changes in the decline of mortality, we first extracted the mortality time index *κ(t)* from the LC model in Eq. (1) and computed the first differences of the series.

4 $$\Delta \kappa_{t} = \kappa_{t} - \kappa_{t - 1}$$

This was motivated by previous research showing that the time series of the time index of the LC model was generally integrated of order one (Coelho and Nunes 2011). This carries the danger of detecting spurious breaks, which could be avoided by using the first differences of the series (Nunes et al. 1995), resulting in the following model:

5 $$\Delta \kappa_{t} = \beta_{0} + \varepsilon_{t}$$

Subsequently, we test the null hypothesis that the change in the time index of the LC model is constant over time against the alternative that there is a single structural change in *β*_*0*_, between 1957 and 2005 using data from 1951 to 2011. The trimming of the first and last six observations in the observation period is a common approach to avoid misleading breaks that are located too close at the boundaries of the data (Harvey et al. 2009). The test involves the computation of an ***F*** statistic for every possible structural change

6 $$F = \frac{{{\text{RSS}} - {\text{ESS}}}}{\text{ESS}} \times \left( {n - 2k} \right)$$

where *n* is the length of the data frame, here *n* = *59*, and *k* the number of explanatory variables in (5), here *k* = *1*, RSS the restricted sum of squares of model (5) and ESS the error sum of squares from the model containing a structural change (Zeileis et al. 2003). The null hypothesis is rejected if the supremum of the ***F*** statistics surpasses the critical values at the significance level of 0.05, provided by Andrews (1993) based on simulations. To date the structural changes, we applied the dynamic programming approach suggested by Bai and Perron (2003), whereby—based on the Bayesian Information Criterion (BIC)—an optimal number of segments and related breakpoints in a time series is identified by examining all possible partitions sequentially. Although this approach is able to identify an optimal number of multiple structural changes, it does not allow to test specific hypotheses unlike the ***F*** test explained above. Therefore, we follow Zeileis et al. (2003) who suggest to first perform an ***F*** test for the existence of *at least* one structural change and second to search for the *optimal number* of breakpoints using the BIC-based procedure of Bai and Perron. All analyses were performed by using the *strucchange* package in *R* (Zeileis et al. 2002; R Core Team 2014).

## Results

Structural changes at the significant level of 0.05 in the central time trend of the LC model were detected in 12 of the 20 countries, representing 60 % of the sample (Table 1). Removing the impact of smoking took away structural changes in 7 of these 12 countries (58.3 %), namely Australia, Austria, Canada, Scotland, England &Wales, Italy and the USA. In the remaining 5 countries, namely Denmark, Ireland, Netherlands, Norway and Sweden, the presence and timing of the detected structural changes was robust to the adjustment for the impact of smoking, except for the Netherlands where the break point was dated at a later point in time (2003 instead of 2002). Thereby, in none of the cases did the procedure of Bai and Perron (2003) select more than one structural change within a country—neither before nor after adjusting for smoking.

**Table 1:** Structural changes in the time index of the Lee–Carter model before and after removing smoking-associated mortality, males at age 0 to 85+

Country	Before adjustment for smoking				After adjustment for smoking			
	Break date	F statistic	*P* value	Change in rate of decline	Break date	F statistic	P value	Change in rate of decline
Australia	1970	13.5	0.01	−0.5		8.1	0.07	
Austria	1983	11.4	0.02	−0.4		6.4	0.16	
Belgium		6.1	0.18			1.2	1.00	
Canada	1995	21.6	0.00	−0.4		8.6	0.06	
Switzerland		8.1	0.08			3.4	0.54	
Denmark	1995	25.3	0.00	−0.7	1995	9.8	0.03	−0.4
Spain		4.3	0.38			1.3	0.99	
Finland		2.9	0.64			2.7	0.68	
France		4.6	0.34			2.5	0.73	
Northern Ireland		7.2	0.11			3.5	0.51	
Scotland	1993	10.3	0.03	−0.4		3.2	0.58	
England &Wales	1985	10.8	0.02	−0.4		4.0	0.43	
Ireland	1999	12.9	0.01	−0.7	1999	9.8	0.03	−0.7
Italy	1983	13.6	0.01	−0.5		3.8	0.47	
Japan		4.7	0.32			6.0	0.19	
Netherlands	2002	24.7	0.00	−0.9	2003	12.3	0.01	−0.7
Norway	1990	18.0	0.00	−0.6	1990	13.7	0.01	−0.5
Portugal		8.0	0.08			7.0	0.12	
Sweden	1988	12.9	0.01	−0.5	1988	9.6	0.04	−0.4
USA	1968	13.7	0.01	−0.3		5.1	0.28	
% of cases with breaks	60 %				25 %			

The detection of possible structural changes was restricted to the years 1957–2005

Generally, the change in the rate of the decline in the time index of the LC model after the structural break—corresponding to change in *β*_*0*_ in (5)—was less pronounced when adjusting for smoking (Table 1). The most striking structural changes were observed in Denmark, Ireland and the Netherlands where the mortality decline accelerated after the change by 0.7, 0.7 and 0.9 units, respectively, which was smaller in the Netherlands, similar in Ireland and much smaller in Denmark after adjustment for the impact of smoking.

**Fig. 1:**
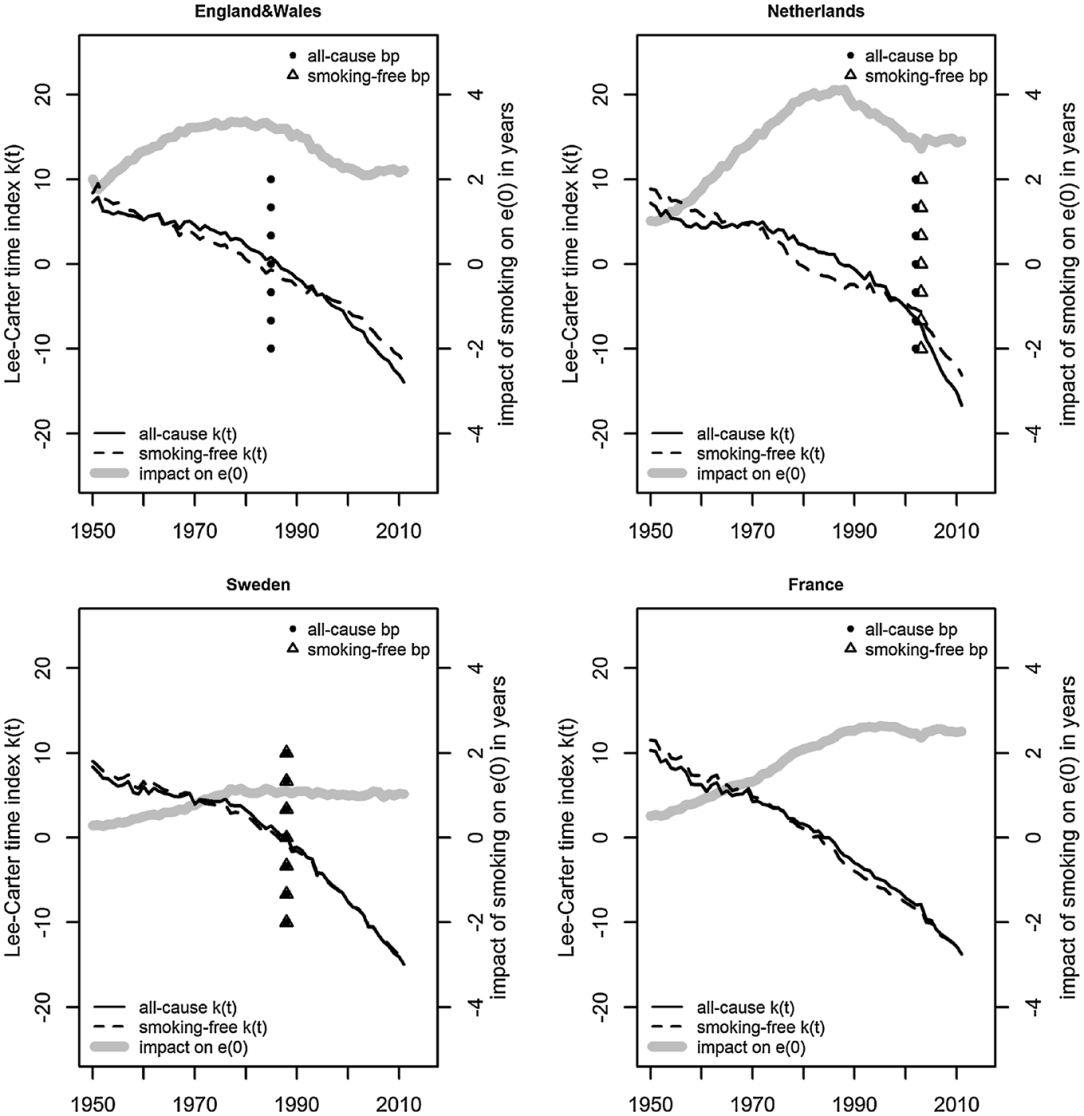
Trends in the series in the time index of the Lee–Carter model before and after removing smoking-associated mortality and the impact of smoking on life expectancy at birth in England & Wales, the Netherlands, Sweden and France, males at age 0 to 85+ *Note* Dots indicate the breakpoint (all-cause breakpoint) of the series in all-cause mortality, while triangles indicate the breakpoint (smoking-free breakpoint) of the series in smoking-free mortality. The detection of possible structural changes was restricted to the years 1957–2005

We have illustrated the impact of smoking on mortality decline for four countries, each representing a different type (Fig. 1): England & Wales as one country, where the impact of smoking explained the structural change; the Netherlands, where after removing the impact of smoking, the timing of the break point changed; Sweden, where adjustment for the impact of smoking did not explain the structural change; and France, where no structural change was detected at all. The graphs also display the different patterns of the impact of smoking on life expectancy at birth. In France, the impact of smoking on life expectancy increased more gradually and less strongly, whereas this was much more pronounced in the Netherlands where the damage of smoking accumulated rapidly to about 4 years loss of life expectancy and subsequently decreased to a loss of about 3 years in 2011.

We have tested the sensitivity of our results with respect to the exclusion of several subgroups that are less effected by the impact of smoking, namely infants up to age 5, youth up to age 35 and elderly above age 85 (Table 2). These checks reveal that at least 44 % and perhaps even up to 78 % of the structural changes are explained by smoking, whereby the number of countries with a break varied between 8 and 12 before and between 2 and 5 after adjusting for smoking. If persons below age 35 were removed from the sample, even 90 % of the countries (18 out of 20) did not exhibit any significant structural changes after adjustment for the impact of smoking.

**Table 2:** The sensitivity of the frequency of detecting breakpoints in 20 OECD countries with respect to the age range used to fit the Lee–Carter model before and after removing smoking-associated mortality

Excluded subgroup(s)	Lower age limit	Upper age limit	Number of detected breaks		Breaks explained by smoking (in pct) (%)
			Before adjusting for smoking	After adjusting for smoking	
None	0	85+	12	5	58
Infants	5	85+	9	3	67
Infants & youth	35	85+	9	2	78
Elderly	0	84	12	5	58
Infants & elderly	5	84	9	5	44
Infants & youth & elderly	35	84	8	4	50

The detection of possible structural changes was restricted to the years 1956–2005

## Discussion

This is the first study to assess the hypothesis that the impact of the tobacco epidemic caused the presence of structural changes in the central time trend of the LC model statistically. Removing the impact of smoking took away the majority of detected structural changes. In the few countries where the structural changes remained, adjusting for smoking attenuated the change in the rate of mortality decline. These results provide solid evidence for the hypothesis that the progression of the smoking epidemic explains structural changes in mortality trends, which is both relevant for the explanation of past mortality trends and for the projection of mortality.

### Evaluation of Data and Methods

First, we evaluate the quality of our data before discussing possible explanations for the results of our study with respect to the applied methods. The data on mortality were obtained from harmonized sources that rely on vital registries and population censuses of high quality (Luy 2011). For the countries included in our study, the information on all-cause mortality and on lung-cancer mortality was virtually complete, so that our results are hardly affected by missing data which is often a problem in cross-country comparisons that cover a longer time frame (Reibling 2013). Although data on causes of death for several decades are often affected by coding changes or improved diagnostic possibilities, this is less the case for lung-cancer deaths that could be identified relatively clearly and were not subject to problematic coding changes (Alter and Carmichael 1996).

In our analysis, we used the time index of the LC model as a central indicator of the trend in mortality decline, as this is most relevant for forecasting life expectancy at birth. Thus, our findings partly depend on the capability of the model to provide a reliable description of the past trends in mortality. It is known that the LC model is not able to capture more complex patterns in mortality trends, such as cohort-driven influences, since it allows only for a trivial correlation structure among death rates (Preston et al. 2014; Renshaw and Haberman 2006). However, by removing the impact of smoking from mortality rates, we accounted for the most important determinant of cohort effects, so that smoking-free mortality trends are certainly less affected by the simplicity of the LC model (Willets 2004). Moreover, the frequency of the detection of breakpoints in mortality trends does not fundamentally change by using models more complex than the LC model (O’Hare and Li 2014b).

Another potential source of bias might be the indirect approach to quantify the impact of smoking on mortality. Building on observed lung-cancer death rates, the PGW approach utilizes a reliable indicator for the cumulative intensity of smoking to estimate the total damage of smoking on mortality. This arrives at results comparable to the more complex approaches that utilize more causes of death associated with smoking to arrive at estimates of smoking-attributable mortality (Bronnum-Hansen and Juel 2000; Rostron 2010). Nevertheless, in common with all other competing approaches, the PGW approach involves a static assumption as the damage of smoking on lung cancer is translated to the damage of smoking on causes other than lung cancer in the same period (Oza et al. 2011). Thus, the trend and magnitude of the impact of smoking on mortality is determined solely by the trend and magnitude of lung-cancer mortality (Preston et al. 2010; Oza et al. 2011). This ignores that the time lag between smoking and cardiovascular causes is likely much shorter and that the time lag between smoking and chronic obstructive pulmonary disease is much longer than the time lag between smoking and lung cancer (Murphy and Di Cesare 2012; Oza et al. 2011). However, more advanced models that took such timing differentials into account arrived at similar estimates of smoking-attributable mortality (Oza et al. 2011).

A particular strength of our analysis is the use of a formal statistical test procedure to decide on the presence of a structural change. Previous articles in the field of population studies usually decided about the presence of such changes solely based on the better fit to the data, e.g., Booth et al. (2002), Vallin and Meslé (2009), Ouellette et al. (2014), bearing the risk of detecting breaks that are in fact random fluctuations (Nunes et al. 1995; Hastie et al. 2008). We tried to minimize that risk by taking first differences of the time series, and by using a sound ***F*** test specifically designed for detecting a single unknown structural change combined with a BIC-based procedure to decide on the number of breaks, which penalizes more complex models to prevent overfitting. This more conservative approach might also explain why we detected fewer breaks than for instance Ouellette et al. (2014), who report changes in 28 of 34 countries (82.3 %) compared to 12 of 20 countries (60 %) in our analysis.

The detection of structural changes in our model was clearly sensitive to the size of the sample with respect to the age range included (Table 2). Ouellette et al. (2014) proposed that the population aged ≤40 years should be excluded from the analysis because, for younger ages, other determinants may be more important, while O’Hare and Li (2014a) argued that the data at higher ages is of less quality and removed observations above age 89 in their analysis. These particular concerns are not confirmed by our sensitivity checks despite the observed variation with respect to the included age range. Smoking explained 58 % of the structural changes in the model with the full age range and 50 % of the structural changes in the model with the most restricted age range (age 35–84). In all versions of the sensitivity check, the effect of smoking was large enough to explain a substantial part of the breakpoints no matter whether unaffected subgroups were excluded or not. This confirms that smoking is by far the most important source of structural changes on mortality—even visible if the analysis was not specifically tailored to ages where smoking is more relevant.

In our study design, we did not include the female subpopulation since there smoking was deemed to be less relevant. We nevertheless replicated our analysis for women and detected in none of the countries a structural change in the rate of mortality decline (results not shown). After adjusting for smoking, a single structural change in mortality trends of Dutch females appeared in 2002 but in none of the other 19 countries.

### Interpretation

Our findings contribute to the literature on mortality forecasting and on determinants of mortality trends in high-income countries.

Changes in the time index of the LC model fitted to smoking-free mortality data were stable between 1951 and 2011 in at least three-fourths of the countries. Thus, for a large number of countries, robust long-term projections taking the whole time series of observations as input are feasible. To avoid misleading projections, it seems advisable to project smoking-associated mortality and non-smoking mortality separately, as proposed by Janssen et al. (2013). This contrasts with alternative solutions that suggested to extrapolate all-cause mortality based on the trends of the most recent stable period only (Li et al. 2011; Booth et al. 2002). Doing so will clearly lead to wrong forecasts, since the *temporary* faster decline in mortality rates due to the diminishing impact of smoking is extrapolated *permanently* into the future. For these reasons, our results are in line with previous work claiming that the development of new projection models, particularly in the actuarial field, should take smoking more seriously than in the past (Peters et al. 2016).

Unfortunately, our results also indicate that the removal of smoking alone cannot solve the problem of irregularities in past mortality trends in all cases (Janssen et al. 2013; Bongaarts 2006). A promising candidate for a factor that could be also relevant to explain sudden changes in mortality trends is the health-care system in high-income countries that is becoming increasingly important for further gains in life expectancy (Bunker 2001); this is discussed in more detail below.

The structural changes in males that occurred more recently were found in countries that underwent major reforms in their health system, i.e., Denmark, Ireland and the Netherlands. In Denmark the so-called heart plan was launched during the mid-1990s and was targeted at reducing cardiovascular mortality, which may explain the detected change from slower to more rapid improvement in 1995 (Christensen et al. 2010). In Ireland, among other measures, free health-care services for those aged ≥70 years were implemented at the turn of the twentieth century, which resulted (for instance) in a dramatic increase in prescriptions for statins (McDaid et al. 2009; Walley et al. 2005). This increase in prescriptions explained a trend break in mortality trends at around 1999 that was replicated in our study (Layte et al. 2011). In the Netherlands, fixed hospital budgets were relaxed in 2001 resulting in a rapid increase in hospital admissions, medical prescriptions and shorter waiting times for elective surgery, particularly among the elderly, probably related to the sudden improvement in life expectancy (Peters et al. 2015a, b; Mackenbach et al. 2011).

Besides Denmark, two other Nordic states exhibited persistent structural changes in males, namely Sweden in 1988 and Norway in 1990. Possibly also in these countries, improved medical care may have contributed to the more rapid decline in mortality. For instance in Sweden, expensive medical treatments such as dialysis and coronary by-pass grafting were applied much more often at the end of the 1980, while this was restricted to younger persons due to informal age limits in the medical system before (Broomé et al. 1994). Due to rapidly growing revenues from the oil sector since the 1970s, Norway became one of the richest countries in the world and exhibited the highest growth rate in per capita health spending among all OECD countries between 1990 and 2001 potentially enabling the more rapid decline in mortality (Johnsen and Bankauskaite 2006). Further research should test the potential impact of improved medical care in these two countries in more detail.

The fact that we detected no structural changes in female mortality trends corresponds with studies showing that smoking was less relevant for mortality trends in the past, since females took up smoking much later in time and to a lesser extent (Thun et al. 2012). However, the rapidly increasing lung-cancer death rates in women should be seen as warning that the stable mortality decline in females should not be taken for granted and that periods with temporary slower improvements are possible.

## Conclusion

The impact of the tobacco epidemic explains the frequent occurrence of structural changes in the time index of the LC model in men to a large extent. Thus, uncovering the underlying reasons of structural changes in the decline of mortality is feasible and should become a standard procedure before mortality projections are obtained. To identify further determinants of the observed breakpoints in mortality series, the impact of health-care policies and innovations in medical treatments on mortality trends should be taken into account more carefully.
